# Buffalo Academy of Medicine

**Published:** 1898-02

**Authors:** Thomas F. Dwyer


### BUFFALO ACADEMY OF MEDICINE.

REPORTED BY THOMAS F. DWYER, M. D., Secretary.

*Quarterly meeting held at the Academy parlors, Palace Arcade, Tuesday evening, January 11, 1898.*

MEETING called to order at 8.45 P. M. by the president, Dr. LUCIEN HOWE. Minutes of last quarterly meeting were read and approved.

The secretary reported the favorable action of the council on the following applicants: Drs. Dennis J. Constantine, H. C. Rooth, Almon H. Cooke, A. W. Bayliss, Albert J. Colton, Frederick M. Boyle, Edgar A. Forsyth, Francis W. McGuire, Martha F. Caul and Edward L. Frost for resident membership, and Drs. C. G. Leo Wolf, Niagara Falls, N. Y., and C. R. Regan, North Tonawanda, N. Y., for nonresident membership. They were ballotted for separately and duly elected fellows of the Academy. The president then stated that he had a very unpleasant duty to perform in announcing the death of one of the prominent members of the Academy, a former chairman of the section of surgery, Dr. William S. Tremaine.

On motion a committee of three was appointed to draft resolutions on the death of Dr. Tremaine. The Chair appointed as such committee Drs. Chauncey P. Smith, William H. Heath and C. C. Wyckoff.

The program of the evening under the auspices of the section of surgery consisted of the following: The parasitic origin of cancer with demonstrations of the organisms and microscopical sections, Dr. Roswell Park; discussed by Dr. Herbert U. Williams and Dr. Woods Hutchinson.

Dr. PARK received a very warm welcome from the members, as was evidenced by the hearty applause of the ninety-six present.

The PRESIDENT, on behalf of the academy, extended to him a welcome on his safe return from abroad very much improved in health. Dr. Park feelingly thanked the president and members of the academy, collectively and individually, for their kind reception.

The following resolutions on the death of Dr. Tremaine were then read by Dr. William H. Heath:

The Buffalo Academy of Medicine deeply deploring the death of Dr. William S. Tremaine, one of its early members, herewith tenders its sincere sympathy to his saddened family, and desires publicly to bear testimony to his character, worth and personality. Intrepid in his aggressiveness for the advancement of his profession and its ethics, to which he contributed more than ordinary, fearless in all that to his light was right, loyal to a degree unexcelled, the craft of medicine has lost not only a faithful worker, but those who knew him, a loyal friend ; this community has lost a valuable life, the poor of it a benefactor whose kindness and consideration were without limit. His deep sense of all obligations was ever uppermost in his mind, and it can be truly said that his early and untimely end was, in no small degree, hastened by his self-sacrificing sense of duty.

It is the sense of this Academy that a copy of the sentiments herein expressed be transmitted to the family, that they be made known to the public through the press, and to the profession at large through the BUFFALO MEDICAL JOURNAL.

WILLIAM H. HEATH,
CHAUNCEY P. SMITH,
C. C. WYCKOFF.

On motion of Dr. ROSWELL PARK, seconded by Dr. William G. Ring, the above memorial was adopted.

Adjourned at 10.45 P. M.

